# Discrepancy of QF-PCR, CMA and karyotyping on a de novo case of mosaic isodicentric Y chromosomes

**DOI:** 10.1186/s13039-018-0413-1

**Published:** 2019-01-09

**Authors:** Yuan Liu, Li Guo, Hanbiao Chen, Jian Lu, Jingjing Hu, Xianzheng Li, Xing Li, Ting Wang, Fengzhen Li, Aihua Yin

**Affiliations:** 1 Prenatal Diagnosis Centre, Guangdong Women and Children Hospital, Guangzhou, 511400 Guangdong China; 2 Maternal and Children Metabolic-Genetic Key Laboratory, Guangdong Women and Children Hospital, Guangzhou, 511400 Guangdong China

**Keywords:** Karyotyping, Isodicentric Y, FISH, CMA, QF-PCR, Prenatal diagnosis

## Abstract

**Background:**

Isodicentric chromosomes are the most frequent structural aberrations of human Y chromosome, and usually present in mosaicism with a 45, X cell line. Several cytogenetic techniques have been used for diagnosing of uncommon abnormal sex chromosome abnormalities in prenatal cases.

**Case presentation:**

A 26-year-old healthy woman was referred to our centre at 24 weeks of gestation age. Ultrasound examination indicated she was pregnant with imbalanced development of twins. Amniocentesis was referred to the patient for further genetic analyses. Quantitative Fluorescent Polymerase Chain Reaction (QF-PCR) indicated the existence of an extra Y chromosome or a structurally abnormal Y chromosome in primary amniotic cells. Chromosome microarray (CMA) analysis based on Comparative Genomic Hybridization (aCGH) platform was performed and identified a 10.1 Mb deletion on Y chromosome in 8-days cultured amniotic cells. Combined with the data of QF-PCR and aCGH, karyotyping and fluorescence in situ hybridization (FISH) revealed a mosaic cell line of 45,X[27]/46,X, idic(Y)(q11.22) [14] in fetus.The karyotyping analysis of cord blood sample was consistent with amniotic cells. The parental karyotypes were normal, which indicated this mosaic case of isodicentric Y (idicY) chromosomes of the fetus was a de novo case.

**Conclusion:**

Several approaches have been used for the detection of numerical and structural chromosomal alterations of on prenatal cases. Our report supported the essential role of incorporating multiple genetic techniques in prenatal diagnosing and genetic counseling of potential complex sex chromosomal rearrangements.

## Background

Chromosome abnormality is one of the leading causes of fetal malformations and early pregnancy loss [1]. Isodicentric chromosomes are the most commonly structural aberrations of human Y chromosome, and often present in mosaicism with a 45,X cell line due to their mitotic instability [2]. Several detection approaches have been used for detecting numerical and structural chromosomal alterations in prenatal examination. FISH and QF-PCR have been applied to detect aneuploidies of chromosomes 21, 18, 13, X and Y for offering rapid results (2–3 days) by using primary cells or tissues [3, 4]. As they are chromosomal probe-dependent, only probe-specific abnormalities could be identified. For years, CMA technology has been proved to be equivalent to karyotype analysis in detection of common aneuploidies [5]. In addition to that, high resolution of CMA favors the detection of micro chromosomal imbalances, which could not be identified by conventional karyotype analysis [6–8]. In prenatal genetic analysis, CMA has been recommended to patient with fetal structural anomalies and/or stillbirth instead of fetal karyotype [4]. On the other hand, the major disadvantages of CMA is imprecise interpretation of variants of unknown significance [4, 5]. Moreover, CMA technology has limitations in detecting balanced chromosomal rearrangements and low levels of mosaicism, which could be identified by conventional karyotyping [8–10]. Despite its time-consuming, labor-intensive manner and limited resolutions, karyotyping still plays critical role in the detection of inherited chromosomal rearrangements in prenatal diagnosis and genetic counseling [4, 11]. This report highlighted the importance of the incorporation of conventional karyotyping and molecular genetic techniques in clinical practice, especially in prenatal diagnosis of uncommon chromosome abnormalities.

## Case presentation

### Clinical report

**Fig. 1:**
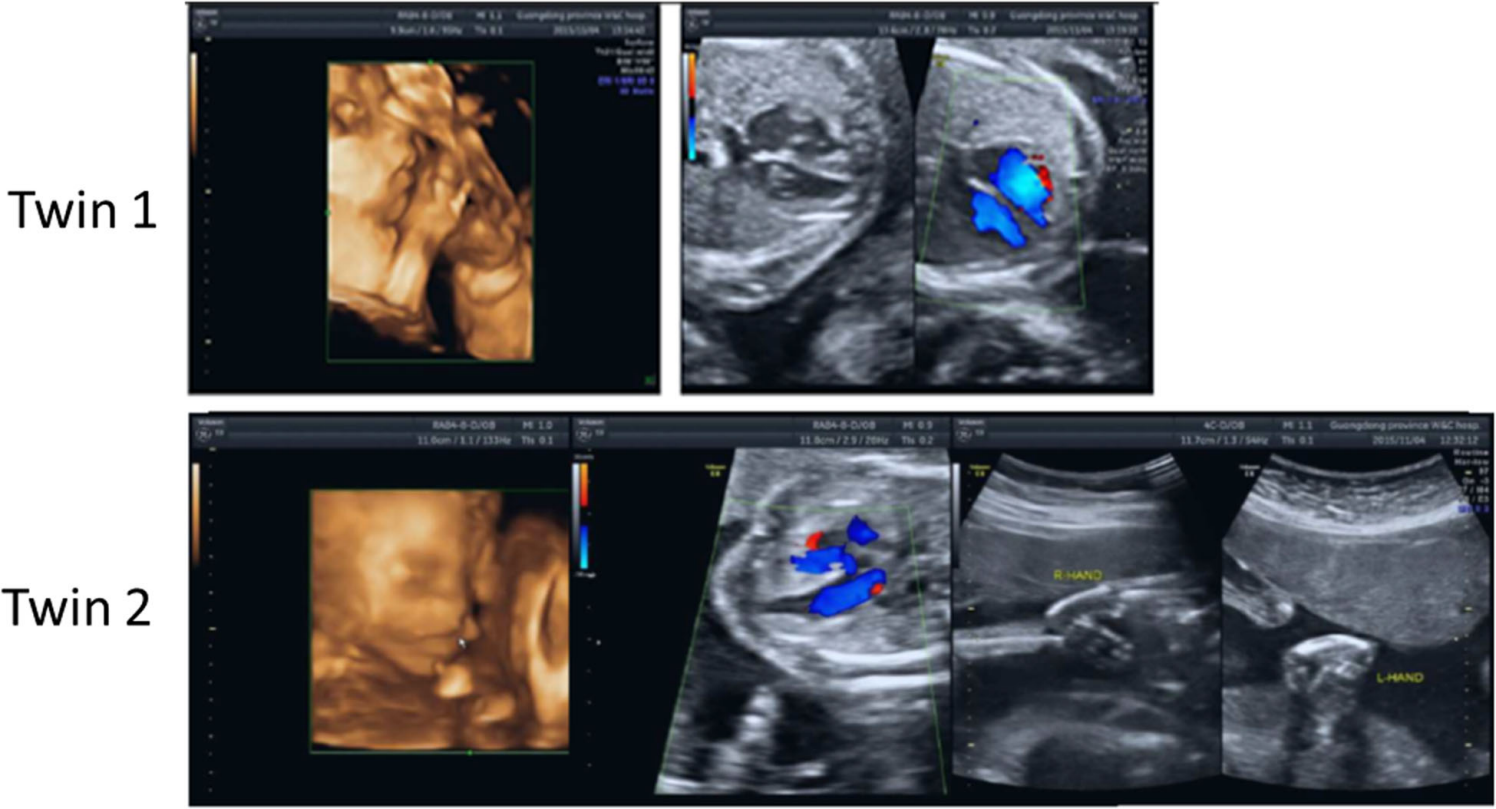
Ultrasound examination of twin 1(up) and twin 2 (down) at the gestation age of 24 weeks

A 26-year-old woman, G1P0A0, was referred to the Medical Genetic Centre of Guangdong Women and Children Hospital for prenatal diagnosis at 24 weeks of gestation due to imbalanced development of twins. The patient’s medical history revealed no remarkable abnormalities. The patient got pregnant naturally and had no family history of twins or multiple births. Fetal ultrasound scans showed a monochorionic diamniotic pregnancy with imbalanced development of twins. Twin 1 presented with normal development of brain, abdomen, skeleton and cardiovascular system. Twin 2 had normal brain and abdomen, with underdeveloped/absent radius, ventricular septal defect, cleft lip and palate (Fig. 1).

Ultrasound parameters for twin 1 (Fig. 1 up): Biparietal diameter (BPD) 59 mm, Head circumference (HC) 223 mm, Abdominal circumference (AC) 198 mm, Femur length (FL) 45 mm, Heart rate (HR) 154/min.

Ultrasound parameters for Twin 2 (Fig. 1 down): BPD 51 mm, HC192mm, AC1 41 mm, FL 35 mm, HR 141/min.

Data from ultrasound examination indicated the imbalanced development of the two fetuses might due to the Twin-to-twin transfusion syndrome (TTTS), but can’t exclude chromosomal abnormalities. After genetic counseling, the couple agreed to receive a diagnostic amniocentesis for the normal fetus (twin 1).

### Cytogenetic analysis

Amniotic cells were cultured in CHANG Medium (CHANG Amnio, Irvine Scientific) for 7–10 days for karyotyping and CMA analysis. Conventional G-banded karyotyping from peripheral blood lymphocytes and cord blood were performed according standard protocols. Array Comparative Genomic Hybridization (aCGH) analysis was performed using Agilent’s 8 × 60 K commercial arrays (Agilent Technologies, CA, USA) and the data was analyzed with AgilentGenomic Workbench Lite Edition 6.5.0.18 software (Agilent Technologies) as described in our previous report [12]. QF-PCR for detecting common chromosome numerical anomalies was carried out using a modified version of previous report [13]. FISH based on cultured amniotic cells was performed by using AneuVysion Multicolor DNA Probe Kit (Abbott Molecular Inc., USA).

## Results

**Fig. 2:**
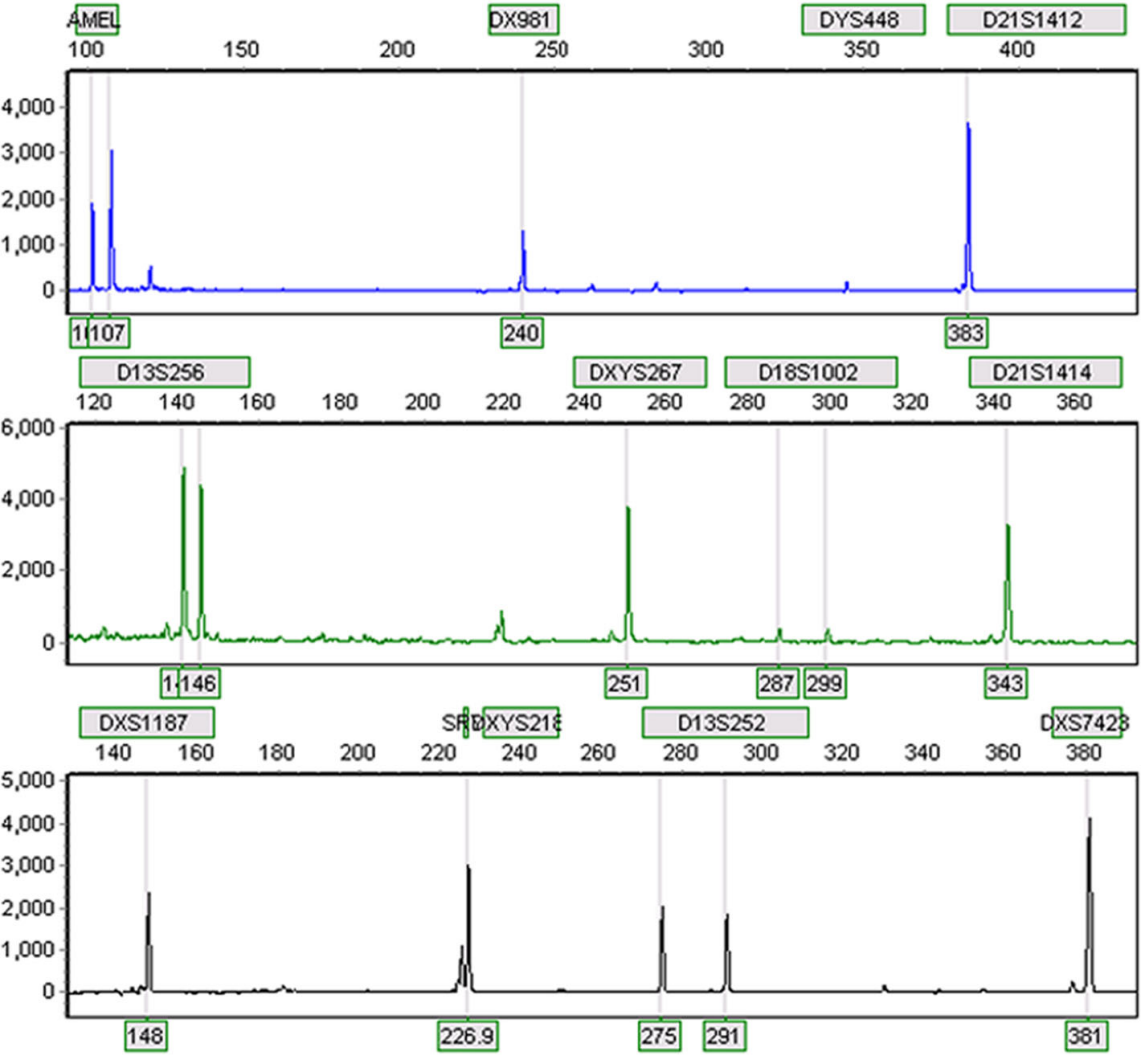
Rapid QF-PCR analysis on uncultured amniotic cells. The analysis indicated the fetus might have an extra Y chromosome or a structurally abnormal Y chromosome

**Fig. 3:**
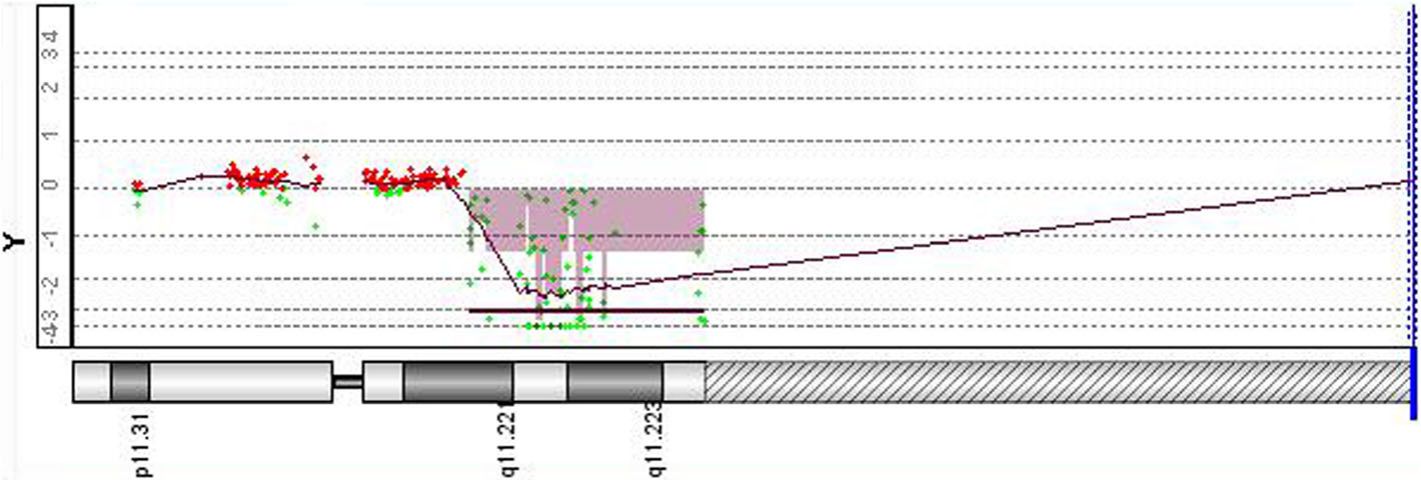
Array-based CGH analysis of cultured amniotic cells. The analysis indicated a 10.1 Mb deletion on Yq11.221-q11.23 region, with the deleted base pair coordinate ranging from 17,073,540–27,176,992 (hg18).

**Fig. 4:**
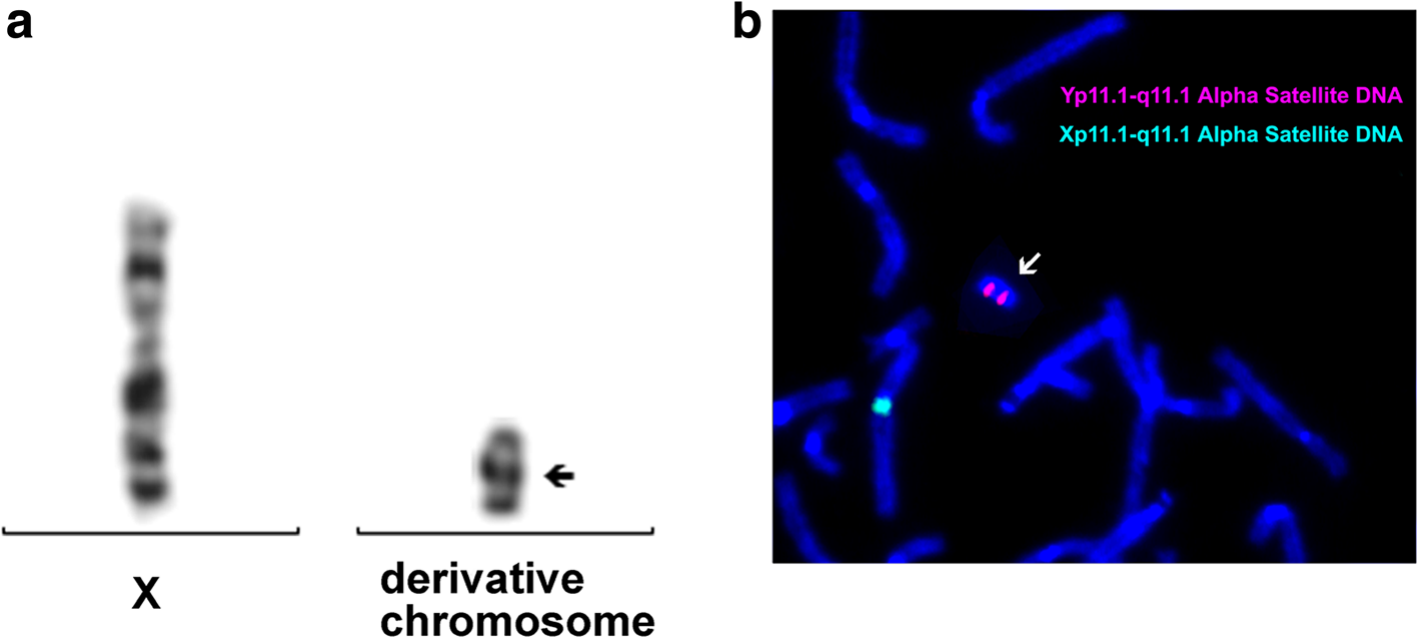
Karyotyping and FISH analysis of cultured amniotic cells. Karyotyping indicated a mosaic of 45,X and 45,X plus an derivative chromosome (**a**). FISH (**b**) was performed using Alpha Satellite DNA probe located in Xp11.1-q11.1 and Yp11.1-q11.1 (AneuVysion Multicolor DNA Probe Kit, Abbott, USA).The data revealed the derivative chromosome was composed of two Y chromosome centromeres (arrow)

Day 3 post amniocentesis, rapid QF-PCR analysis indicated the existence of an extra Y chromosome or structural abnormality on Y chromosome (Fig. 2). Array-CGH analysis of 8-days cultured amniotic cells detected a 10.1 Mb deletion on Yq11.221-q11.23 (Fig. 3). On the other hand, karyotyping analysis of 8-days cultured amniotic cells demonstrated a mosaic of 45,X and 45,X plus an derivative chromosome (Fig. 4a), in 27 and 14 counted cell colonies respectively. To further identify the source of that derivative chromosome, amniotic cells were sub-cultured and FISH analysis was performed. Specific probes for centromeres of X chromosome (Xp11.1-q11.1) and Y chromosome (Yp11.1-q11.1) were applied in FISH.As shown in Fig. 4b, two Y centromeres were found on the derivative chromosome. Thus, the final result of karyotyping was mos 45,X[27]/46,X, idic(Y)(q11.22, 14].

After genetic counseling, the pregnancy was terminated at 30 weeks of gestation. The cord blood of the twins and the peripheral blood of the parents were collected and delivered to laboratory for karyotyping. The results demonstrated normal karyotypes of both parents. The father had a normal size of Y chromosome and no loss or abnormal of Y chromosomes were identified by counting 100 metaphase. Karyotyping of the cultured cord blood lymphocytes showed that both fetuses possessed mosaic karyotype of 45,X/46,X, idic(Y). The mosaic level of 45,X in Twin 1 and Twin 2 were 5 and 10% respectively.

## Discussion and conclusions

To find out the cause of the imbalance development of the twins, QF-PCR, CMA, Karyotyping and FISH were applied to rule out chromosomal abnormalities. However, none of them led to a precise diagnosis. By using primary amniotic cells, rapid QF-PCR analysis showed the presence of SRY and AMEL genes which means the fetus might be male. Meanwhile, the AMEL peaks were present in 0.64:1 ratio which indicated that the fetus might have an extra Y chromosome. However, DYS448 marker locating in the AZFc region of the long arm of Y chromosome didn’t show any peak. All of these data indicated the fetus possessed abnormal Y chromosomes (Fig. 2). By using 8-days cultured amniotic cells, aCGH analysis only detected a 10.1 Mb deletion on Yq11.221-q11.23 without any duplication on Y chromosome or other chromosomes (Fig. 3). In addition to that, karyotyping of cultured amniotic cells showed a mosaic of 45, X and 45, X plus a derivative chromosome (Fig. 4a). Taken together, we hypothesized that the derivative chromosome found in karyotyping was a rearranged Y chromosome. To confirm this, FISH was performed on sub-cultured amniotic cells. Two Y chromosome centromeres on the derivative chromosome were then identified, which indicated that the derivative chromosome could result from the fusion of Y chromosome after breakage on Yq11.2 region (Fig. 4b). IdicY chromosomes are formed during the process of spermatogenesis through homologous crossing over between opposite palindrome arms on sister chromatids [14]. As reported previously, Yq11.2 region is the common breakage point of idic Y and most of the cases were de novo [2, 15], which was consistent with normal karyotype of the father of twins in this case.

The clinical manifestation of the patient with idicY chromosome ranges from spermatogenic failure to Turner syndrome, depending on the gene loss of Y chromosome and the mosaic level of 45,X [2, 15–17]. After assessing the risk, the couple decided to terminate the pregnancy at 30 weeks of gestation. Karyotyping on cord blood from aborted fetus showed that Twin1 had lower mosaic level of 45,X than Twin 2 (5% vs 10%),which might contribute to the imbalance development of the twins. Moreover, as the patient was monochorionic diamniotic pregnancy, TTTS should be considered as the other significant reason resulting in the imbalance development of the twins.

As reported previously, idic Y chromosome often presents as a mosaic with 45,X cell line due to their mitotic instability [2, 16, 18]. In present case, the variable ratio between 45,X and 46,X,idic(Y) in primary cells and 8-days-cultured cells might lead to the inconsistent implications of QF-PCR, aCGH and karyotyping. After 8 days culturing, the number of 45,X cells was almost as twice as that of 46,X, idic(Y) cells, for which reason aCGH analysis failed to find any duplication but only identified a deletion on Y chromosomes.

In review of this case, the difference of cultured and uncultured amniotic cells might result in the discrepancy of those cytogenetic techniques. Our report demonstrates that the incorporation of multiple genetic techniques was essential for prenatal diagnosis and genetic counseling, especially when an uncommon Y chromosome aberration was noted.

## Abbreviations

aCGH: Array Comparative Genomic Hybridization
CMA: Chromosome microarray
FISH: Fluorescence in situ hybridization
QF-PCR: Quantitative Fluorescent Polymerase Chain Reaction
TTTS: Twin-to-twin transfusion syndrome
